# Cognitive Stimulation and Activity-Dependent Myelination: Oligodendroglial Mechanisms Linking Neural Activity and Brain Plasticity

**DOI:** 10.3390/ijms27083603

**Published:** 2026-04-18

**Authors:** Jordana Mariane Neyra Chauca, Maclovia Vázquez VanDyck, Ana Lilia Guerrero Oseguera, Catalina Meneses Ramírez, Alexis Didier Gutiérrez Escobar, Iván Peña Orozco, Maria Belen Ramirez Sanchez

**Affiliations:** 1 Facultad de Medicina, Universidad Autónoma de Guadalajara, Guadalajara 45129, Jalisco, Mexico; 2 Facultad de Medicina, Universidad Villa Rica, Boca del Rio 94299, Veracruz, Mexico; 3 Facultad de Medicina, Instituto de Estudios Superiores de Chiapas, Tuxtla Gutiérrez 29049, Chiapas, Mexico; 4 Facultad de Medicina, Universidad Autónoma del Estado de Hidalgo, Pachuca 42000, Hidalgo, Mexico; pe304679@uaeh.edu.mx; 5 Facultad de Estudios Superiores Iztacala, Universidad Nacional Autónoma de Mexico, Tlalnepantla de Baz 54090, Estado de Mexico, Mexico

**Keywords:** activity-dependent myelination, oligodendrocyte lineage cells, white matter plasticity, neuron–glia communication, cognitive stimulation, neuroplasticity, experience-dependent brain remodeling

## Abstract

The capacity of the brain to adapt to experience has long been associated with synaptic plasticity; however, recent evidence demonstrates that experience-driven neural activity also modulates white matter organization through dynamic regulation of oligodendrocyte lineage cells and myelination. Activity-dependent myelination has emerged as a complementary form of neuroplasticity that contributes to circuit efficiency, temporal coordination, and cognitive function. This review aims to examine the neurobiological mechanisms linking cognitive stimulation and activity-dependent neuronal signaling with oligodendroglial dynamics and adaptive myelination. A narrative review of experimental and translational studies was conducted, focusing on evidence from animal models and human research exploring neuron–oligodendroglia interactions, neurotransmitter-mediated signaling, learning paradigms, physical exercise, and neuromodulatory interventions relevant to myelination and brain plasticity. Accumulating evidence indicates that cognitive stimulation, learning, and physical activity modulate neuronal firing patterns and neurotransmitter release, influencing oligodendrocyte precursor cell proliferation, differentiation, and myelin remodeling. Neurotransmitters such as glutamate, GABA, dopamine, and acetylcholine play key roles in neuron–oligodendroglia communication, largely through calcium-dependent intracellular signaling pathways. These mechanisms have been associated with experience-dependent circuit refinement across motor, cognitive, and stress-related paradigms. Rather than implying direct clinical effects, this review highlights oligodendroglial plasticity as a biologically plausible substrate through which cognitive and behavioral experiences may influence adaptive myelination and white matter integrity. Understanding these mechanisms provides a conceptual framework for future research exploring non-pharmacological approaches to modulate brain plasticity at the level of myelin.

## 1. Introduction

The capacity of the central nervous system to adapt to experience is a fundamental property underlying learning, memory, and behavioral flexibility. For decades, this adaptive potential has been primarily attributed to synaptic plasticity, including changes in synaptic strength, dendritic spine remodeling, and activity-dependent modulation of neuronal networks. While these mechanisms remain central to our understanding of brain plasticity, growing evidence suggests that experience-driven neural activity also induces structural and functional changes beyond the synapse, particularly within white matter circuits [1].

In recent years, oligodendrocytes and myelin have emerged as dynamic contributors to neural plasticity rather than passive structural elements [2]. Oligodendrocyte lineage cells respond to neuronal activity through multiple signaling pathways, enabling adaptive regulation of myelination in response to experience. This process, commonly referred to as activity-dependent myelination, has been shown to influence axonal conduction velocity, temporal synchronization of neural circuits, and network efficiency—features that are critical for cognitive processing and learning [3].

Experimental studies have demonstrated that learning paradigms, environmental enrichment, and physical exercise can modulate oligodendrocyte precursor cell proliferation, differentiation, and myelin remodeling across multiple brain regions [4]. These findings challenge the traditional view of myelin as a static structure formed predominantly during early development and instead position oligodendroglial plasticity as an experience-sensitive mechanism that persists throughout life. Importantly, these adaptive myelin changes appear to be tightly coupled to neuronal firing patterns and neurotransmitter release, underscoring the bidirectional communication between neurons and oligodendroglia [5].

At the molecular level, neuron–oligodendroglia interactions are mediated by a range of neurotransmitters and neuromodulators, including glutamate, GABA, dopamine, and acetylcholine [6]. Oligodendrocyte lineage cells express functional receptors for these signaling molecules, allowing them to sense neuronal activity and translate it into intracellular calcium-dependent signaling cascades that regulate differentiation and myelin formation [7]. Through these mechanisms, cognitive and behavioral experiences have the potential to influence white matter organization in a manner that complements synaptic plasticity.

Despite the increasing recognition of activity-dependent myelination as a key component of brain plasticity, the integration of oligodendroglial mechanisms into models of cognitive stimulation and learning remains incomplete. Most frameworks addressing cognitive plasticity continue to emphasize neuronal and synaptic adaptations, often overlooking the contribution of white matter dynamics [8]. A comprehensive understanding of how cognitive stimulation influences brain function, therefore, requires consideration of oligodendrocyte biology and myelin remodeling as integral components of experience-dependent neural adaptation.

The present narrative review aims to synthesize current experimental and translational evidence linking cognitive stimulation, neuronal activity, and oligodendroglial dynamics. By focusing on mechanisms of activity-dependent myelination and neuron–glia communication, this review seeks to provide a conceptual framework for understanding how cognitive and behavioral experiences may shape white matter plasticity and contribute to functional circuit optimization.

## 2. Methods

This manuscript was developed as a narrative review aimed at synthesizing current experimental and translational evidence on the relationship between cognitive stimulation, neuronal activity, and activity-dependent myelination mediated by oligodendrocyte lineage cells. Given the mechanistic and conceptual nature of the topic, a narrative approach was selected to allow integrative discussion of heterogeneous findings across cellular, systems-level, and behavioral studies.

A comprehensive literature search was conducted using major scientific databases, including PubMed, Scopus, and Web of Science, covering the period from January 2000 to December 2025. Searches were performed using combinations of keywords related to oligodendrocyte biology and myelin plasticity, such as oligodendrocytes, oligodendrocyte precursor cells, activity-dependent myelination, white matter plasticity, neuron–glia interaction, cognitive stimulation, learning, environmental enrichment, stress, and physical exercise. Reference lists of relevant review articles and primary studies were also screened to identify additional pertinent publications.

The review primarily prioritized in vivo mammalian experimental studies that directly examined neuron–oligodendroglia communication, neurotransmitter- and neuromodulator-mediated signaling, and experience-dependent changes in myelination. In vitro studies (OPC/oligodendrocyte cultures) and non-mammalian models (e.g., zebrafish) were included selectively when they provided mechanistic insights directly relevant to activity-dependent myelination in mammalian systems. Human studies, including neuroimaging and translational research, were included when they provided complementary evidence linking cognitive or behavioral experiences to white matter adaptations.

Studies were selected based on their relevance to the central themes of the review: (i) mechanisms of activity-dependent myelination, (ii) neurotransmitter and neuromodulatory control of oligodendrocyte lineage dynamics, (iii) effects of cognitive stimulation and learning paradigms on myelin remodeling, and (iv) modulation of myelination by stress, physical exercise, and environmental context. Due to the narrative nature of the review, no quantitative synthesis or meta-analysis was performed.

The selected literature was analyzed qualitatively to identify convergent mechanisms, recurring patterns, and conceptual frameworks linking neuronal activity to oligodendroglial plasticity. When conflicting or controversial findings were identified, priority was given to convergent evidence from independent laboratories, methodological differences across studies (experimental models, species, age, stimulation paradigms, and analytical approaches) were considered, and relevant discrepancies were explicitly discussed in the Discussion section rather than excluding such studies. Findings were integrated across studies to construct a cohesive model of experience-dependent myelination, while also highlighting methodological limitations and gaps in current knowledge. This approach was chosen to facilitate critical interpretation and to support a mechanistic discussion of oligodendrocyte-mediated plasticity within broader models of brain function.

## 3. Oligodendrocytes and Myelin as Dynamic Elements of Brain Plasticity

Traditionally, oligodendrocytes and myelin were regarded as static structural components of the central nervous system, primarily responsible for insulating axons and ensuring efficient saltatory conduction. Myelination was long considered a developmental process that reached completion early in life, with limited capacity for remodeling in adulthood [9,10]. However, accumulating experimental evidence has challenged this view, demonstrating that oligodendrocyte lineage cells retain a remarkable degree of plasticity and responsiveness to neuronal activity across the lifespan [11,12].

Oligodendrocyte precursor cells (OPCs) persist in the adult brain and continuously survey the neural environment. These cells are capable of proliferating, migrating, and differentiating into mature myelinating oligodendrocytes in response to changes in neuronal activity [13,14]. Importantly, OPCs form synapse-like contacts with neurons and express functional receptors for a variety of neurotransmitters, positioning them as active participants in neural circuit dynamics rather than passive support cells [15,16].

Activity-dependent myelination has emerged as a key mechanism through which experience influences white matter architecture. Experimental studies have shown that increases in neuronal firing, whether induced by learning paradigms, motor training, or environmental enrichment, are associated with region-specific changes in myelin thickness, internode length, and oligodendrocyte number [17,18,19]. These structural modifications have functional consequences, as they can fine-tune axonal conduction velocity and improve temporal coordination within neural networks [20].

Beyond structural insulation, myelin plasticity contributes to information processing by regulating the timing and synchronization of action potential propagation. Small changes in myelin properties can significantly alter signal transmission delays, thereby influencing network oscillations and the precise temporal relationships required for cognitive functions such as learning and memory [21]. In this context, myelination represents a form of long-term plasticity that complements synaptic modifications and supports stable circuit optimization.

At the cellular level, the dynamic behavior of oligodendrocytes is tightly coupled to neuronal activity patterns. Neuronal firing influences oligodendroglial behavior not only through direct synaptic-like signaling but also via activity-dependent release of neurotransmitters and neuromodulators into the extracellular space [22].

Importantly, oligodendroglial plasticity appears to be highly context-dependent, varying across brain regions and functional circuits. White matter tracts associated with learning, motor coordination, and higher cognitive processing show particularly pronounced activity-dependent myelin adaptations.

Together, these findings support a reconceptualization of oligodendrocytes and myelin as dynamic and experience-sensitive elements of brain plasticity. Rather than serving solely as structural support, oligodendroglial lineage cells actively contribute to the adaptive refinement of neural circuits, providing a biological substrate through which experience and cognitive stimulation can shape white matter organization.

## 4. Neuron–Oligodendroglia Communication and Activity-Dependent Signaling

The interaction between neurons and oligodendrocyte lineage cells constitutes a critical interface through which neural activity is translated into adaptive structural modifications of white matter. Rather than acting as passive support cells, oligodendrocyte precursor cells (OPCs) actively sense neuronal firing through multiple signaling modalities, enabling tight coupling between circuit activity and myelin remodeling [23,24].

OPCs establish specialized contacts with axons that share structural and functional features with synapses. Through these neuron–OPC junctions, neuronal activity directly modulates oligodendroglial behavior. Electrophysiological and imaging studies have shown that action potential firing elicits rapid OPC responses, including membrane depolarization and intracellular calcium transients, underscoring their capacity to decode activity patterns within local neural circuits [25,26,27].

Neurotransmitter-mediated signaling plays a central role in neuron–oligodendroglia communication. Both OPCs and mature oligodendrocytes express functional receptors for excitatory and inhibitory neurotransmitters, allowing them to respond to synaptic and extrasynaptic neurotransmitter release [28,29]. Glutamatergic signaling, in particular, has been shown to regulate OPC proliferation and differentiation through activation of ionotropic and metabotropic glutamate receptors. Activity-dependent glutamate release from axons, therefore, acts as a key instructive signal linking neuronal firing to myelination [30,31].

In parallel, GABAergic signaling contributes to the fine-tuning of oligodendroglial responses. GABA receptors expressed on OPCs modulate membrane potential and calcium dynamics, influencing the timing of differentiation and myelin formation. The balance between excitatory and inhibitory inputs appears to be a critical determinant of oligodendrocyte lineage progression, suggesting that circuit-level activity patterns shape myelination in a context-dependent manner [32].

Beyond classical neurotransmitters, neuromodulatory systems exert broader control over oligodendroglial dynamics. Dopamine and acetylcholine—closely associated with attention, learning, and behavioral state—have been implicated in the regulation of OPC behavior and myelin plasticity. Unlike fast synaptic transmission, these neuromodulators operate over wider spatial and temporal scales, providing a mechanism through which global brain states such as arousal, motivation, and cognitive engagement may influence white matter remodeling [33,34].

A unifying feature of these signaling pathways is the involvement of intracellular calcium signaling. Calcium transients within oligodendrocyte lineage cells serve as integrative signals linking receptor activation to downstream transcriptional programs and cytoskeletal remodeling required for differentiation and myelin production. The frequency, amplitude, and spatial distribution of calcium signals appear to encode information about neuronal activity patterns, enabling oligodendrocytes to respond selectively to functionally relevant circuit dynamics [26,28,32,33,35].

**Figure 1:**
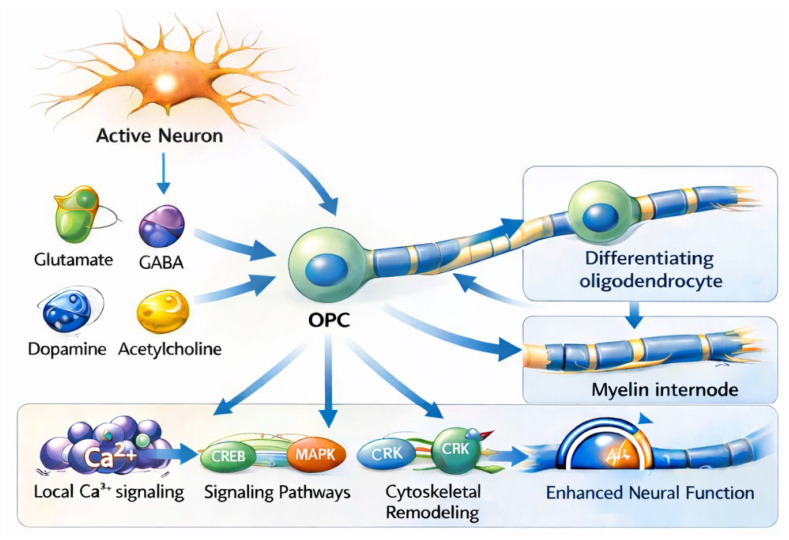
Activity-dependent myelination mediated by oligodendrocyte lineage cells. Neurotransmitter release from active neurons (glutamate, GABA, dopamine, and acetylcholine) engages oligodendrocyte precursor cells (OPCs), triggering intracellular signaling cascades including local Ca^2+^ signaling, CREB, MAPK, and CRK pathways, as well as cytoskeletal remodeling. These processes promote OPC differentiation and the formation of myelin internodes, contributing to enhanced neural function. Background shaded areas delineate functional domains or biological processes, while arrows indicate the directionality of activity-dependent signaling and modulatory interactions. Bidirectional arrows represent reciprocal feedback between myelin remodeling and neural function.

Collectively, these mechanisms position neuron–oligodendroglia communication as a central mediator of activity-dependent myelination. By integrating local synaptic inputs and global neuromodulatory cues, oligodendrocyte lineage cells translate cognitive and behavioral experiences into adaptive changes in white matter structure. This bidirectional interaction highlights the importance of incorporating glial signaling pathways into contemporary models of neural plasticity [36]. These multilevel signaling interactions are integrated into a conceptual model of activity-dependent myelination, illustrating how neuronal activity is translated into oligodendroglial differentiation, myelin remodeling, and network optimization (Figure 1).

### Synaptic and Extrasynaptic Signaling to OPCs and Oligodendrocytes: Implications for Myelin Plasticity

OPCs receive classical synaptic inputs, predominantly mediated by glutamate and GABA, enabling them to directly sense patterned neuronal activity and local circuit dynamics [7,8,9,11,12]. These neuron–OPC synapses provide temporally precise information about axonal firing, positioning OPCs as active sensors of activity patterns within neural networks. In contrast, mature oligodendrocytes exhibit limited synaptic connectivity and primarily respond to extrasynaptic and neuromodulatory signals within the extracellular milieu [8,23].

Neurotransmitters and neuromodulators—including glutamate, GABA, dopamine, acetylcholine, norepinephrine, serotonin, and ATP/adenosine—modulate distinct aspects of oligodendrocyte lineage behavior. These signals regulate OPC proliferation, migration, and differentiation, as well as the stabilization and remodeling of myelin sheaths along active axons [6,7,23,24,37,38]. The integration of fast synaptic transmission with slower neuromodulatory cues provides a multiscale regulatory framework through which neuronal activity shapes myelin plasticity in a context-dependent manner [26,39,40].

Differential responsiveness between OPCs and mature oligodendrocytes suggests complementary roles in activity-dependent myelin plasticity. OPCs are particularly sensitive to synaptic activity patterns that instruct lineage progression and spatial targeting of myelination, whereas mature oligodendrocytes integrate neuromodulatory signals to adjust sheath formation, internode length, and myelin thickness along functionally engaged axons [24,41,42,43]. These cellular distinctions translate into distinct functional outputs of myelin plasticity, including de novo myelin sheath formation, remodeling of pre-existing internodes, and fine-tuning of axonal conduction velocity and network synchronization [43,44,45].

Collectively, these mechanisms highlight that activity-dependent myelination emerges from the coordinated action of synaptic and extrasynaptic communication pathways rather than a single signaling modality [23,26,40].

All glial cells depicted correspond to oligodendrocyte lineage cells, including oligodendrocyte precursor cells (OPCs), differentiating oligodendrocytes, and mature myelinating oligodendrocytes. Neuronal activity promotes the release of neurotransmitters and neuromodulators (e.g., glutamate, GABA, dopamine, acetylcholine), which are sensed by OPCs and oligodendrocytes. This signaling induces localized intracellular Ca^2+^ transients and activates downstream pathways (e.g., CREB, MAPK), leading to oligodendrocyte differentiation and activity-dependent myelin internode formation, encompassing both de novo myelin sheath generation and remodeling of pre-existing internodes along active axons. The schematic represents a dynamic, bidirectional process with activity-dependent feedback loops rather than a strictly linear sequence of events, contributing to optimized conduction velocity, temporal coordination of neural signaling, and network function.

## 5. Role of Neurotransmitters and Neuromodulators in Activity-Dependent Myelination

Neurotransmitters and neuromodulators constitute key molecular signals through which neuronal activity influences oligodendrocyte lineage dynamics and adaptive myelination. Oligodendrocyte precursor cells (OPCs) and mature oligodendrocytes express a diverse repertoire of receptors for classical neurotransmitters, neuromodulators, and purinergic signals, enabling them to detect and integrate activity-dependent cues beyond direct synaptic transmission [37,46].

Glutamate represents one of the primary neurotransmitters mediating neuron–oligodendroglia communication. OPCs express functional AMPA, NMDA, and metabotropic glutamate receptors, through which glutamatergic signaling regulates OPC proliferation, differentiation, and survival [47,48]. Activity-dependent glutamate release from axons has been shown to promote myelin formation and remodeling, supporting the notion that excitatory neurotransmission provides instructive signals that couple neuronal firing patterns to adaptive myelination [49,50].

Inhibitory signaling also plays a critical modulatory role. GABA receptors expressed on OPCs influence membrane potential and intracellular calcium dynamics, thereby regulating the timing of differentiation and myelin production [41]. Rather than acting as a simple inhibitory constraint, GABAergic signaling appears to contribute to the fine-tuning of oligodendrocyte lineage progression, helping to balance excitatory-driven myelination and prevent aberrant or excessive myelin formation [42]. This excitatory–inhibitory interplay highlights the importance of circuit-level activity patterns in shaping myelination outcomes.

Beyond fast synaptic transmission, neuromodulatory systems exert broader and more sustained effects on oligodendroglial behavior. Dopamine, acetylcholine, norepinephrine, and serotonin—neurotransmitters closely linked to attention, motivation, learning, and behavioral state—have been implicated in the regulation of OPC proliferation, differentiation, and myelin plasticity [38,51]. These neuromodulators typically operate over extended spatial and temporal scales, providing a mechanism by which global brain states can influence white matter remodeling in a context-dependent manner.

Dopaminergic signaling, in particular, has been associated with region-specific effects on oligodendrocyte lineage cells, especially within circuits involved in reward, motivation, and motor control [52]. Acetylcholine has similarly been linked to experience-dependent myelination, potentially coupling attentional demands and cognitive engagement to adaptive changes in white matter structure [53]. Together, these systems suggest that myelination is sensitive not only to local neuronal firing but also to broader neuromodulatory contexts that reflect behavioral relevance.

A common downstream feature of neurotransmitter and neuromodulator signaling in oligodendrocyte lineage cells is the activation of intracellular calcium pathways. Calcium signaling serves as a central integrative mechanism through which diverse extracellular cues converge to regulate cytoskeletal dynamics, gene expression, and myelin sheath formation. Differences in calcium signal amplitude, frequency, and spatial distribution are thought to encode distinct aspects of neuronal activity, enabling oligodendrocytes to respond selectively to specific functional demands within neural circuits [39].

Collectively, neurotransmitters and neuromodulators provide a multilayered signaling framework that links neuronal activity to oligodendroglial plasticity. Through the integration of fast synaptic signals and slower, state-dependent neuromodulatory inputs, oligodendrocyte lineage cells are able to translate complex patterns of neural activity into adaptive myelination. This mechanism reinforces the concept of myelin as a dynamic and experience-sensitive component of neural plasticity rather than a static structural feature [54]. The major neurotransmitters and neuromodulators involved in neuron–oligodendroglia communication, together with their principal receptors, intracellular signaling pathways, and functional effects on oligodendrocyte lineage cells, are summarized in Table 1.

## 6. Cognitive Stimulation and Experience-Dependent Myelination: Main Experimental Paradigms

Cognitive stimulation encompasses a broad range of experiences engaging learning, attention, memory, executive function, and sensorimotor integration, all of which are characterized by sustained and patterned neuronal activity across distributed brain networks [55,56]. Beyond synaptic modifications, experience-driven neuronal activity shapes neural circuits through complementary mechanisms involving oligodendrocyte lineage dynamics and adaptive myelin remodeling [57]. To provide a more focused framework, we discuss three main experimental paradigms that have been most consistently linked to activity-dependent myelination: (i) skill learning and training paradigms, (ii) multimodal stimulation and environmental enrichment, and (iii) sensory and stress-related modulation of experience-dependent myelination.

### 6.1. Skill Learning and Training Paradigms

Motor skill learning, spatial navigation, and associative learning paradigms provide robust evidence that repeated, task-specific neuronal activity drives oligodendroglial plasticity in functionally relevant circuits. Experimental studies demonstrate that learning is accompanied by increased OPC proliferation, accelerated differentiation into mature oligodendrocytes, and region-specific remodeling of myelin sheaths within task-engaged networks [40,58,59,60]. These oligodendroglial changes correlate with improvements in behavioral performance and learning consolidation, supporting a functional contribution of adaptive myelination to experience-dependent circuit optimization [61].

### 6.2. Multimodal Stimulation and Environmental Enrichment

Environmental enrichment paradigms, which integrate cognitive, social, sensory, and motor stimulation, induce sustained increases in neuronal activity and network engagement. Such multimodal experiences promote oligodendrocyte lineage dynamics, including enhanced OPC differentiation and myelin remodeling in cortical and limbic regions involved in cognition and memory [40,58,59]. These structural adaptations parallel improvements in cognitive flexibility, learning efficiency, and resilience to environmental challenges, indicating that enriched environments potentiate experience-dependent myelin plasticity through coordinated circuit activation.

### 6.3. Sensory Experience and Stress-Related Modulation of Myelination

Manipulations of sensory input and exposure to chronic stress provide additional insight into the context dependence of experience-driven myelination. Sensory deprivation or enhanced stimulation modulates OPC maturation and region-specific myelin formation within sensory pathways, highlighting the sensitivity of oligodendroglial dynamics to patterned neuronal activity [60,61]. In contrast, chronic stress exposure is associated with altered oligodendrocyte morphology, dysregulated myelin integrity, and transcriptional changes within prefrontal and limbic circuits, which parallel behavioral vulnerability and impaired cognitive-emotional regulation. Together, these findings indicate that experience-dependent myelination is bidirectionally modulated by the quality and valence of environmental inputs.

Representative experimental paradigms linking experience to activity-dependent myelination, including affected brain regions, methodological approaches, and functional relevance, are summarized in Table 2.

Experience-driven myelination is mediated in part by activity-dependent modulation of neuronal firing patterns and neurotransmitter release. Coordinated neuronal activity provides instructive signals that promote oligodendrocyte differentiation and myelin formation along active axons, thereby fine-tuning conduction velocity, enhancing temporal precision, and stabilizing functionally relevant circuits that support learning and memory [44,62].

Importantly, experience-dependent oligodendroglial responses occur within defined temporal windows. Evidence from animal models indicates that OPC proliferation and early oligodendrogenesis are rapidly induced during initial phases of learning, whereas subsequent myelin remodeling contributes to longer-term stabilization of circuit changes and behavioral retention [40,44,60,63,64]. This temporal dissociation supports a model in which adaptive myelination complements faster synaptic plasticity processes to consolidate experience-dependent network reorganization.

Rather than implying direct therapeutic effects, these observations support the concept of cognitive stimulation as a biologically plausible driver of oligodendroglial plasticity. By engaging specific neural circuits in a sustained and behaviorally relevant manner, experience-dependent neuronal activity biases myelination toward functionally engaged pathways, positioning adaptive myelination as an integral component of cognitive neuroplasticity [45,65].

## 7. Stress, Exercise, and Environmental Modulation of Myelination

Environmental and behavioral factors exert a significant influence on activity-dependent myelination by shaping neuronal activity patterns and neuromodulatory states. Among these factors, stress and physical exercise represent particularly relevant modulators, as both can induce widespread and sustained changes in neural circuit function. The impact of these experiences on oligodendrocyte lineage cells highlights the sensitivity of myelination processes to the broader physiological and environmental context [66].

Exposure to stress, especially during early developmental periods, has been shown to alter oligodendrocyte maturation and myelin organization across multiple brain regions [67]. Animal models of social isolation and early-life adversity demonstrate reductions in myelin thickness, altered oligodendrocyte morphology, and changes in the expression of myelin-related genes within prefrontal and limbic circuits [68]. These alterations appear to reflect disruptions in experience-dependent myelination rather than primary defects in oligodendrocyte generation, underscoring the role of environmental input in shaping white matter development.

In contrast, physical exercise is generally associated with beneficial effects on brain structure and function, including modulation of oligodendrocyte lineage dynamics [69]. Aerobic exercise has been shown to increase oligodendrogenesis and promote myelin remodeling within hippocampal and cortical circuits, potentially through activity-dependent and neuromodulatory mechanisms [70]. Importantly, exercise represents a mild physiological stressor that engages stress-response pathways in a regulated manner, distinguishing its effects from those of chronic or adverse stress.

The differential impact of stress and exercise on myelination may be mediated, in part, by glucocorticoid signaling and hypothalamic–pituitary–adrenal (HPA) axis activity [37,71]. Sustained elevation of stress hormones has been associated with impaired oligodendrocyte differentiation and reduced myelin formation, whereas adaptive modulation of glucocorticoid signaling during exercise may support neural plasticity [72]. These findings suggest that the qualitative nature of stress exposure—rather than stress per se—plays a critical role in determining myelination outcomes.

Environmental enrichment provides an additional framework for understanding how combined cognitive, social, and sensorimotor experiences influence myelination. Enriched environments consistently produce more pronounced oligodendroglial and myelin adaptations than isolated stimuli, indicating that convergent activity across multiple domains enhances experience-dependent white matter plasticity [73]. Such environments may amplify circuit engagement and neuromodulatory signaling, thereby promoting coordinated myelin remodeling across functionally relevant networks.

Together, these observations highlight stress, exercise, and environmental context as powerful modulators of activity-dependent myelination. Rather than exerting uniform effects, these factors interact dynamically with neuronal activity and neuromodulatory systems to shape oligodendrocyte behavior in a circuit- and context-dependent manner. This perspective reinforces the view of myelination as a plastic and experience-sensitive process that integrates biological, behavioral, and environmental signals [34,59,74].

## 8. Discussion

The evidence synthesized in this review supports a reconceptualization of myelination as a dynamic and experience-sensitive process that actively contributes to neural plasticity. Rather than serving solely as a developmental or structural feature, myelin emerges as a modifiable component of neural circuits that responds to neuronal activity, cognitive engagement, and environmental context. Integrating oligodendroglial dynamics into models of brain plasticity, therefore, expands traditional synapse-centered frameworks toward a more comprehensive view of circuit adaptation [74,75].

A central finding across experimental paradigms is the close coupling between neuronal firing patterns and oligodendrocyte lineage behavior. Activity-dependent signaling enables oligodendrocyte precursor cells to selectively myelinate functionally engaged axons, suggesting that myelin remodeling may reinforce behaviorally relevant pathways and stabilize emerging circuit configurations. In this context, adaptive myelination appears well positioned to complement synaptic plasticity by refining conduction velocity and enhancing temporal coordination within neural networks [43].

Neurotransmitter and neuromodulatory systems further shape these processes across multiple spatial and temporal scales. While fast synaptic transmission provides localized, circuit-specific cues, neuromodulators convey information about global brain states such as attention, motivation, and arousal. Oligodendrocyte lineage cells integrate these diverse signals through intracellular calcium-dependent mechanisms, allowing myelin plasticity to reflect both local activity and broader behavioral relevance [76].

Cognitive stimulation and learning paradigms consistently demonstrate region-specific effects on oligodendrogenesis and myelin remodeling, reinforcing the notion that myelination is selectively tuned to circuit demand rather than uniformly distributed across the brain. Evidence suggests that oligodendrocyte responses often occur during early phases of learning, indicating that myelination may contribute to the consolidation of circuit changes initiated by faster synaptic mechanisms. Environmental factors, including stress and physical exercise, further modulate these effects, emphasizing the sensitivity of myelination to physiological and contextual variables [77].

Despite these advances, several important limitations must be acknowledged. Much of the current evidence for activity-dependent myelination is derived from animal models, in which neuronal activity and oligodendrocyte dynamics can be precisely manipulated. Translating these findings to the human brain remains challenging due to species differences, circuit complexity, and methodological constraints. In human studies, white matter adaptations are typically inferred from indirect imaging measures that lack cellular resolution, limiting direct assessment of oligodendrocyte behavior and myelin remodeling [78].

Additional limitations arise from the heterogeneity of cognitive and environmental experiences. Cognitive stimulation encompasses diverse tasks that engage multiple neural systems, making it difficult to isolate specific activity patterns responsible for observed myelin changes. Moreover, individual variability in age, genetic background, and neuromodulatory state likely influences oligodendroglial responsiveness to activity, complicating interpretation across studies.

Importantly, activity-dependent myelination should not be viewed as a deterministic or uniformly beneficial outcome of cognitive or behavioral engagement. Current evidence does not support direct clinical extrapolation, nor does it allow prediction of functional outcomes based solely on presumed myelin changes. Overinterpretation of these mechanisms risks oversimplifying the complexity of oligodendrocyte biology and experience-dependent plasticity [40,64,68,79].

**Figure 2:**
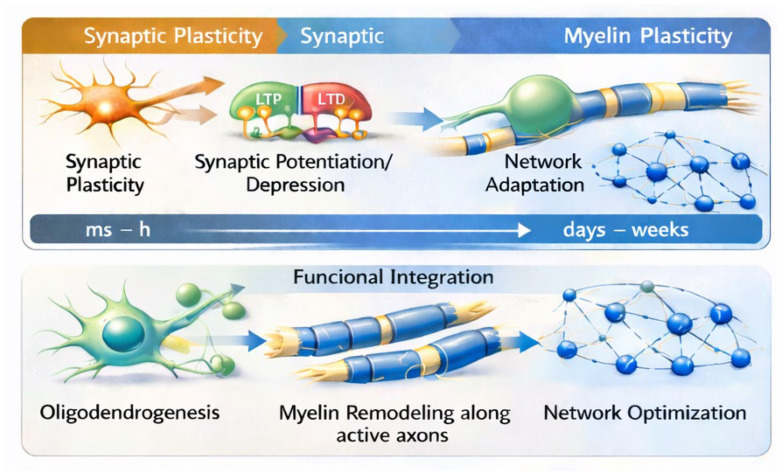
Complementary temporal scales of synaptic and myelin plasticity in neural circuit adaptation. Activity-dependent synaptic plasticity, including long-term potentiation (LTP) and long-term depression (LTD), operates on rapid timescales (milliseconds to hours) and drives early network-level adaptations. In parallel, oligodendrogenesis and experience-dependent myelin remodeling along active axons unfold over longer timescales (days to weeks), contributing to network optimization and functional integration. Background color gradients indicate distinct plasticity domains (synaptic plasticity vs. myelin plasticity) and temporal progression, while arrows represent the directionality of activity-dependent propagation from synaptic changes to network-level reorganization and myelin remodeling.

Overall, incorporating oligodendroglial plasticity into contemporary models of brain function provides a more integrative framework for understanding how neuronal activity and experience shape neural circuits. Recognizing both the potential and the limitations of current evidence will be essential for guiding future research aimed at clarifying the role of adaptive myelination in cognition and behavior across the lifespan [79]. To summarize the multiscale dynamics of neural plasticity discussed in this review, Figure 2 illustrates the complementary temporal organization of synaptic and myelin plasticity and their convergence in long-term circuit optimization.

All glial cells depicted correspond to oligodendrocyte lineage cells, including OPCs and mature oligodendrocytes. The upper panel illustrates synaptic plasticity mechanisms, such as long-term potentiation and long-term depression (LTP/LTD), which operate over milliseconds to hours and enable rapid modulation of synaptic efficacy and network adaptation. The lower panel depicts oligodendrogenesis and activity-dependent myelin remodeling along active axons, processes that unfold over days to weeks and support longer-term structural optimization, stabilization of neural circuits, and sustained functional integration.

## 9. Conclusions

Activity-dependent myelination has emerged as a fundamental and dynamic component of experience-dependent neuroplasticity, expanding traditional views that have historically emphasized synaptic mechanisms as the primary drivers of circuit adaptation [40,74,75]. Accumulating evidence indicates that oligodendrocyte lineage cells actively sense neuronal activity and integrate synaptic and neuromodulatory signals to regulate myelin formation and remodeling in a circuit-specific manner [49,50,54]. Through these processes, myelination contributes not only to axonal insulation but also to the fine-tuning of temporal coordination, signal transmission efficiency, and network synchronization [43,44,45].

Cognitive stimulation, learning, physical activity, and environmental context shape myelination by modulating neuronal firing patterns and global brain states [55,58,66,70]. Rather than acting uniformly across the brain, adaptive myelination appears selectively biased toward functionally engaged circuits, supporting the stabilization and optimization of neural pathways that are repeatedly recruited by experience [59,60,61]. This selective nature of myelin remodeling provides a biologically plausible mechanism through which transient cognitive and behavioral experiences may lead to enduring structural adaptations within white matter [56,57,73].

Importantly, myelin plasticity operates in close interaction with synaptic mechanisms, suggesting a complementary relationship between fast, reversible synaptic changes and slower, more persistent modifications of white matter architecture [15,43,44]. Within this framework, synaptic plasticity may initiate circuit reorganization, while adaptive myelination contributes to the consolidation and long-term maintenance of these changes [40,74]. Recognizing this interplay offers a more integrative understanding of how neural circuits adapt across multiple timescales [75,80].

Beyond its role in learning and memory, activity-dependent myelination may influence broader aspects of brain function, including network efficiency, cognitive flexibility, and resilience to environmental challenges [46,65,77]. Viewing myelin as a malleable and experience-sensitive element of neural circuits shifts the conceptualization of white matter from a passive substrate to an active participant in brain plasticity [66,75,79].

Together, the evidence reviewed supports a conceptual model in which oligodendroglial plasticity represents an essential, yet often underappreciated, layer of neural adaptation [54,74,80]. Incorporating myelin dynamics into contemporary theories of brain function provides a more comprehensive framework for understanding how cognitive and environmental experiences shape neural systems across the lifespan and highlights promising directions for future research into the cellular mechanisms underlying cognitive neuroplasticity [40,75,79,80].

## Figures and Tables

**Table 1:** Neurotransmitter and neuromodulator regulation of oligodendroglial plasticity.

Neurotransmitter/Neuromodulator	Receptors Expressed in OPCs and Oligodendrocytes	Main Intracellular Signaling Pathways	Functional Effects on Oligodendroglial Lineage	Representative Experimental Evidence
Glutamate	AMPA, NMDA, mGluR5	Ca^2+^ influx, MAPK/ERK, CREB activation	Promotes OPC proliferation and differentiation; activity-dependent myelin formation and sheath elongation	In vivo rodent models; two-photon imaging
GABA	GABA-A, GABA-B	Membrane hyperpolarization; Ca^2+^ modulation	Regulates timing of OPC differentiation and maturation; balances excitatory-driven myelination	Slice electrophysiology; in vivo imaging
Dopamine	D1-like and D2-like receptors	cAMP/PKA signaling; transcriptional modulation	Region-specific modulation of oligodendrogenesis and adaptive myelination in learning- and reward-related circuits	Behavioral paradigms; pharmacological studies
Acetylcholine	Muscarinic receptors (M1–M5)	PLC/IP3-mediated Ca^2+^ signaling	Enhances OPC differentiation and myelin remodeling during attentional and cognitive engagement	In vivo models; optogenetic modulation
Norepinephrine (Noradrenaline)	Adrenergic receptors (α1, α2, β)	cAMP/PKA; Ca^2+^-dependent signaling	Modulates OPC differentiation and activity-dependent myelin plasticity in arousal- and learning-related circuits	In vivo rodent models; pharmacological neuromodulation
Serotonin (5-HT)	5-HT receptor subtypes (e.g., 5-HT1, 5-HT2 families)	GPCR signaling; Ca^2+^ modulation	Regulates OPC proliferative state and maturation; context-dependent modulation of myelin plasticity	In vivo and in vitro studies; pharmacological modulation
ATP/Adenosine	P2Y, A1/A2 receptors	Purinergic signaling; Ca^2+^ oscillations	Regulates OPC migration, proliferation, and differentiation in response to neuronal activity	Cell culture and in vivo studies

**Table 2:** Experimental paradigms linking experience to activity-dependent myelination and white matter plasticity.

Experimental Paradigm	Brain Regions Primarily Involved	Observed Myelin or Oligodendroglial Adaptation	Methodological Approach	Functional Relevance
Motor skill learning	Primary motor cortex, corpus callosum	Increased oligodendrogenesis, enhanced myelin thickness and internode remodeling	Two-photon microscopy, genetic lineage tracing	Motor learning consolidation
Environmental enrichment	Hippocampus, prefrontal cortex	Increased OPC differentiation and region-specific myelin remodeling	Histology, BrdU labeling, transcriptomics	Cognitive flexibility and memory
Aerobic physical exercise	Distributed white matter tracts	Structural remodeling of myelinated pathways and increased oligodendrocyte activity	MRI-DTI, immunohistochemistry	Neuroplasticity and cognitive resilience
Chronic stress exposure	Prefrontal cortex, limbic circuits	Reduced myelin integrity, altered oligodendrocyte morphology and gene expression	Electron microscopy, molecular profiling	Vulnerability to cognitive and emotional dysregulation
Cognitive training (human studies)	Association networks	Experience-dependent white matter plasticity	Longitudinal neuroimaging	Performance optimization and learning

## Data Availability

Not applicable. The original contributions presented in this study are included in the article. Further inquiries can be directed to the corresponding authors.

## References

[1] Fields R.D. (2015). A new mechanism of nervous system plasticity: Activity-dependent myelination. Nat. Rev. Neurosci..

[2] Mount C.W., Monje M. (2017). Wrapped to Adapt: Experience-Dependent Myelination. Neuron.

[3] McKenzie I.A., Ohayon D., Li H., de Faria J.P., Emery B., Tohyama K., Richardson W.D. (2014). Motor skill learning requires active central myelination. Science.

[4] Xiao L., Ohayon D., McKenzie I.A., Gibson E.M., Wedel M., Costa R.M., Hsieh J., Richardson W.D., Barres B.A., Chan J.R. (2016). Rapid production of new oligodendrocytes is required in the earliest stages of motor-skill learning. Nat. Neurosci..

[5] Hughes E.G., Orthmann-Murphy J.L., Langseth A.J., Bergles D.E. (2018). Myelin remodeling through experience-dependent oligodendrogenesis in the adult somatosensory cortex. Nat. Neurosci..

[6] Gibson E.M., Purger D., Mount C.W., Goldstein A.K., Lin G.L., Wood L.S., Inema I., Miller S.E., Bieri G., Zuchero J.B. (2014). Neuronal activity promotes oligodendrogenesis and adaptive myelination in the mammalian brain. Science.

[7] Mensch S., Baraban M., Almeida R., Czopka T., Ausborn J., El Manira A., Lyons D.A. (2015). Synaptic vesicle release regulates myelin sheath number of individual oligodendrocytes in vivo. Nat. Neurosci..

[8] Wake H., Ortiz F.C., Woo D.H., Lee P.R., Angulo M.C., Fields R.D. (2015). Nonsynaptic junctions on myelinating glia promote preferential myelination of electrically active axons. Nat. Commun..

[9] Bergles D.E., Jabs R., Steinhäuser C. (2010). Neuron-glia synapses in the brain. Brain Res. Rev..

[10] Hamilton N., Vayro S., Kirchhoff F., Verkhratsky A., Robbins J., McCarthy K.D. (2008). Mechanisms of ATP- and glutamate-mediated calcium signaling in white matter astrocytes. Glia.

[11] Kukley M., Kiladze M., Tognatta R., Schramm J., Fiehler J., Kirchhoff F. (2008). Glial cells are born with synapses. FASEB J..

[12] Lin S.C., Bergles D.E. (2002). Physiological characteristics of NG2-expressing glial cells. J. Neurocytol..

[13] Rinholm J.E., Hamilton N.B., Kessaris N., Richardson W.D., Bergersen L.H., Attwell D. (2011). Regulation of oligodendrocyte development and myelination by glucose and lactate. J. Neurosci..

[14] Scholz J., Klein M.C., Behrens T.E., Johansen-Berg H. (2009). Training induces changes in white-matter architecture. J. Neurosci..

[15] Zatorre R.J., Fields R.D., Johansen-Berg H. (2012). Plasticity in gray and white: Neuroimaging changes in brain structure during learning. Nat. Neurosci..

[16] Makinodan M., Rosen K.M., Ito S., Corfas G. (2012). A critical period for social experience-dependent oligodendrocyte maturation and myelination. Science.

[17] Liu J., Dietz K., DeLoyht J.M., Goldman S.A., Kocsis J.D., Casaccia P. (2012). Impaired adult myelination in the prefrontal cortex of socially isolated mice. Nat. Neurosci..

[18] Maugeri G., D’Agata V., Musumeci G. (2023). Role of exercise in the brain: Focus on oligodendrocytes and remyelination. Neural Regen. Res..

[19] Voss M.W., Nagamatsu L.S., Liu-Ambrose T., Kramer A.F. (2011). Exercise, brain, and cognition across the life span. J. Appl. Physiol..

[20] Sampaio-Baptista C., Johansen-Berg H. (2017). White Matter Plasticity in the Adult Brain. Neuron.

[21] Sampaio-Baptista C., Khrapitchev A.A., Foxley S., Schlaghecken F., Scholz J., Jbabdi S., DeLuca G.C., Miller K.L., Taylor A., Thomas A. (2013). Motor skill learning induces changes in white matter microstructure and myelination. J. Neurosci..

[22] Fields R.D. (2008). White matter in learning, cognition and psychiatric disorders. Trends Neurosci..

[23] Monje M. (2018). Myelin Plasticity and Nervous System Function. Annu. Rev. Neurosci..

[24] Hughes A.N., Appel B. (2019). Oligodendrocytes express synaptic proteins that modulate myelin sheath formation. Nat. Commun..

[25] Malar D.S., Thitilertdecha P., Ruckvongacheep K.S., Brimson S., Tencomnao T., Brimson J.M. (2023). Targeting Sigma Receptors for the Treatment of Neurodegenerative and Neurodevelopmental Disorders. CNS Drugs.

[26] Saab A.S., Nave K.A. (2017). Myelin dynamics: Protecting and shaping neuronal functions. Curr. Opin. Neurobiol..

[27] Krasnow A.M., Attwell D. (2016). NMDA Receptors: Power Switches for Oligodendrocytes. Neuron.

[28] Spitzer S.O., Sitnikov S., Kamen Y., Krzyzanowska A., Schneider K., Horn M., Höft S., Schäfer M.K.E., Möbius W., Nave K.-A. (2019). Oligodendrocyte Progenitor Cells Become Regionally Diverse and Heterogeneous with Age. Neuron.

[29] Orthmann-Murphy J., Call C.L., Molina-Castro G.C., Geer L.Y., Williamson J.M., Pannullo S.C., Chan J.R. (2020). Remyelination alters the pattern of myelin in the cerebral cortex. eLife.

[30] Saab A.S., Tzvetanova I.D., Nave K.A. (2013). The role of myelin and oligodendrocytes in axonal energy metabolism. Curr. Opin. Neurobiol..

[31] Draganski B., Gaser C., Busch V., Schuierer G., Bogdahn U., May A. (2004). Neuroplasticity: Changes in grey matter induced by training. Nature.

[32] Hofstetter S., Tavor I., Tzur Moryosef S., Assaf Y. (2013). Short-term learning induces white matter plasticity in the fornix. J. Neurosci..

[33] Taubert M., Draganski B., Anwander A., Müller K., Horstmann A., Villringer A. (2010). Dynamic properties of human brain structure: Learning-related changes in cortical areas and associated fiber connections. J. Neurosci..

[34] Adams K.L., Riparini G., Banerjee P., Breur M., Bugiani M., Gallo V. (2020). Endothelin-1 signaling maintains glial progenitor proliferation in the postnatal subventricular zone. Nat. Commun..

[35] Ainger K., Avossa D., Morgan F., Hill S.J., Barry C., Barbarese E., Carson J.H. (1993). Transport and localization of exogenous myelin basic protein mRNA microinjected into oligodendrocytes. J. Cell Biol..

[36] Lövdén M., Wenger E., Mårtensson J., Lindenberger U., Bäckman L. (2013). Structural brain plasticity in adult learning and development. Neurosci. Biobehav. Rev..

[37] De La Fuente A.G., Lange S., Silva M.E., Gonzalez G.A., Tempfer H., van Wijngaarden P., Pringle N.P., Miron V.E., Franklin R.J.M. (2017). Pericytes stimulate oligodendrocyte progenitor cell differentiation during CNS remyelination. Cell Rep..

[38] Nishiyama A., Shimizu T., Sherafat A., Richardson W.D. (2021). Life-long oligodendrocyte development and plasticity. Semin. Cell Dev. Biol..

[39] Noori R., Park D., Griffiths J.D., Bhattacharya P., Tewarie P., van Dellen E., Stam C.J. (2020). Activity-dependent myelination: A glial mechanism of oscillatory self-organization in large-scale brain networks. Proc. Natl. Acad. Sci. USA.

[40] Xin W., Chan J.R. (2020). Myelin plasticity: Sculpting circuits in learning and memory. Nat. Rev. Neurosci..

[41] Bechler M.E., Swire M., Ffrench-Constant C. (2018). Intrinsic and adaptive myelination-A sequential mechanism for smart wiring in the brain. Dev. Neurobiol..

[42] Iyer M., Kantarci H., Cooper M.H., Snaidero N., Möbius W., Nave K.-A. (2024). Oligodendrocyte calcium signaling promotes actin-dependent myelin sheath extension. Nat. Commun..

[43] Almeida R.G., Czopka T., Ffrench-Constant C., Lyons D.A. (2011). Individual axons regulate the myelinating potential of single oligodendrocytes in vivo. Development.

[44] Hill R.A., Li A.M., Grutzendler J. (2018). Lifelong cortical myelin plasticity and age-related degeneration in the live mammalian brain. Nat. Neurosci..

[45] Pajevic S., Basser P.J., Fields R.D. (2014). Role of myelin plasticity in oscillations and synchrony of neuronal activity. Neuroscience.

[46] Khelfaoui H., Ibaceta-Gonzalez C., Angulo M.C. (2024). Functional myelin in cognition and neurodevelopmental disorders. Cell. Mol. Life Sci..

[47] Ye L., Gastaldi V.D., Curto Y., Wildenburg A.-F., Yu X., Hindermann M., Eggert S., Ronnenberg A., Wang Q., Butt U.J. (2025). Transcriptional dynamics of the oligodendrocyte lineage and its regulation by the brain erythropoietin system. Nat. Commun..

[48] Caldwell M., Ayo-Jibunoh V., Mendoza J.C., Muthukumaraswamy S.D., Harris J.J., Attwell D., Richardson W.D. (2023). Axo-glial interactions between midbrain dopamine neurons and oligodendrocyte lineage cells in the anterior corpus callosum. Brain Struct. Funct..

[49] Chen T.J., Kula B., Nagy B., Barzan R., Gall A., Kukley M. (2018). In Vivo Regulation of Oligodendrocyte Precursor Cell Proliferation and Differentiation by the AMPA-Receptor Subunit GluA2. Cell Rep..

[50] Geraghty A.C., Gibson E.M., Ghanem R.A., Greene J.J., Ocampo A., Goldstein A.K., Ni L., Yang T., Marton R.M., Paşca S.P. (2019). Loss of Adaptive Myelination Contributes to Methotrexate Chemotherapy-Related Cognitive Impairment. Neuron.

[51] Choe Y., Huynh T., Pleasure S.J. (2014). Migration of oligodendrocyte progenitor cells is controlled by transforming growth factor β family proteins during corticogenesis. J. Neurosci..

[52] Bergles D.E., Richardson W.D. (2015). Oligodendrocyte Development and Plasticity. Cold Spring Harb. Perspect. Biol..

[53] Rangel-Gomez M., Alberini C.M., Deneen B., Arenkiel B.R., McKnight S.L., Eisch A.J. (2024). Neuron-Glial Interactions: Implications for Plasticity, Behavior, and Cognition. J. Neurosci..

[54] Stedehouder J., Kushner S.A. (2017). Myelination of parvalbumin interneurons: A parsimonious locus of pathophysiological convergence in schizophrenia. Mol. Psychiatry.

[55] Tomassy G.S., Berger D.R., Chen H.H., Kasthuri N., Hayworth K.J., Vercelli A., Seung H.S., Lichtman J.W. (2014). Distinct profiles of myelin distribution along single axons of pyramidal neurons in the neocortex. Science.

[56] Yeung M.S., Zdunek S., Bergmann O., Bernard S., Salehpour M., Alkass K., Perl S., Tisdale J., Possnert G., Brundin L. (2014). Dynamics of oligodendrocyte generation and myelination in the human brain. Cell.

[57] Gao Z.K., Shen X.Y., Han Y., Guo Y.S., Yuan M., Bi X. (2022). Enriched Environment Effects on Myelination of the Central Nervous System: Role of Glial Cells. Neural Plast..

[58] Emery B., Lu Q.R. (2015). Transcriptional and Epigenetic Regulation of Oligodendrocyte Development and Myelination in the Central Nervous System. Cold Spring Harb. Perspect. Biol..

[59] Arnett H.A., Mason J., Marino M., Suzuki K., Matsushima G.K., Ting J.P.Y. (2001). TNFα promotes proliferation of oligodendrocyte progenitors and remyelination. Nat. Neurosci..

[60] Almeida R.G., Pan S., Cole K.L.H., Williamson J.M., Jäkel S., Koch S., Tischbirek C.H. (2018). Myelination of Neuronal Cell Bodies when Myelin Supply Exceeds Axonal Demand. Curr. Biol..

[61] Mossink B., Negwer M., Schubert D., Nadif Kasri N. (2021). The emerging role of chromatin remodelers in neurodevelopmental disorders: A developmental perspective. Cell. Mol. Life Sci..

[62] Jensen S.K., Yong V.W. (2016). Activity-Dependent and Experience-Driven Myelination Provide New Directions for the Management of Multiple Sclerosis. Trends Neurosci..

[63] Fields R.D., Woo D.H., Basser P.J. (2015). Glial Regulation of the Neuronal Connectome through Local and Long-Distant Communication. Neuron.

[64] Nave K.A., Werner H.B. (2014). Myelination of the nervous system: Mechanisms and functions. Annu. Rev. Cell Dev. Biol..

[65] Arancibia-Carcamo I.L., Attwell D. (2014). The node of Ranvier in CNS pathology. Acta Neuropathol..

[66] Chang K.J., Redmond S.A., Chan J.R. (2016). Remodeling myelination: Implications for mechanisms of neural plasticity. Nat. Neurosci..

[67] Lazari A., Lipp I. (2021). Can MRI measure myelin? Systematic review, qualitative assessment, and meta-analysis of studies validating microstructural imaging with myelin histology. Neuroimage.

[68] Wei X., Gunter T.C., Adamson H., Friederici A.D., Skeide M.A., Flöel A. (2024). White matter plasticity during second language learning within and across hemispheres. Proc. Natl. Acad. Sci. USA.

[69] de Lange A.G., Bråthen A.C., Grydeland H., Sexton C., Johansen-Berg H., Walhovd K.B., Fjell A.M. (2016). White matter integrity as a marker for cognitive plasticity in aging. Neurobiol. Aging.

[70] Bennett I.J., Madden D.J. (2014). Disconnected aging: Cerebral white matter integrity and age-related differences in cognition. Neuroscience.

[71] Seidlitz J., Nadig A., Liu S., Bethlehem R.A.I., Vértes P.E., Morgan S.E., Váša F., Romero-Garcia R., Lalonde F.M., Clasen L.S. (2020). Transcriptomic and cellular decoding of regional brain vulnerability to neurogenetic disorders. Nat. Commun..

[72] van Tilborg E., de Theije C.G.M., van Hal M., Wagenaar N., de Vries H.E., Dijkhuizen R.M., Heijnen C.J., Benders M.J.N.L., Nijboer C.H.A. (2018). Origin and dynamics of oligodendrocytes in the developing brain: Implications for perinatal white matter injury. Glia.

[73] Young K.M., Psachoulia K., Tripathi R.B., Dunn S.J., Cossell L., Attwell D., Tohyama K., Richardson W.D. (2013). Oligodendrocyte dynamics in the healthy adult CNS: Evidence for myelin remodeling. Neuron.

[74] Barateiro A., Fernandes A. (2014). Temporal oligodendrocyte lineage progression: In vitro models of proliferation, differentiation and myelination. Biochim. Biophys. Acta.

[75] Badimon A., Strasburger H.J., Ayata P., Chen X., Nair A., Ikegami A., Hwang P., Chan A.T., Graves S.M., Uweru J.O. (2020). Negative feedback control of neuronal activity by microglia. Nature.

[76] Berghoff S.A., Spieth L., Sun T., Hosang L., Schlaphoff L., Depp C., Düking T., Winchenbach J., Neuber J., Ewers D. (2021). Microglia facilitate repair of demyelinated lesions via post-squalene sterol synthesis. Nat. Neurosci..

[77] Timmler S., Simons M. (2019). Grey matter myelination. Glia.

[78] Micheva K.D., Wolman D., Mensh B.D., Pax E., Buchanan J., Smith S.J. (2016). A large fraction of neocortical myelin ensheathes axons of local inhibitory neurons. Elife.

[79] Camargo N., Goudriaan A., Van Deijk A.-L.F., Otte W.M., Brouwers J.F., Lodder H., Gutmann D.H., Nave K.-A., Dijkhuizen R.M., Mansvelder H.D. (2017). Oligodendroglial myelination requires astrocyte-derived lipids. PLoS Biol..

[80] Mitew S., Gobius I., Fenlon L.R., McDougall S.J., Hawkes D., Xing Y.L., Bujalka H., Gundlach A.L., Richards L.J., Kilpatrick T.J. (2018). Pharmacogenetic stimulation of neuronal activity increases myelination in an axon-specific manner. Nat. Commun..

